# Trends in Health Expectancies by Late-Life Disadvantage: The Cognitive Function and Ageing Studies

**DOI:** 10.1093/geroni/igaa057.2197

**Published:** 2020-12-16

**Authors:** Holly Bennett, Andrew Kingston, Gemma Spiers, Louise Robinson, Clare Bambra, Carol Brayne, Fiona Matthews, Carol Jagger

**Affiliations:** 1 Newcastle University, Newcastle upon Tyne, England, United Kingdom; 2 Cambridge Institute of Public Health, University of Cambridge, Cambridge, England, United Kingdom

## Abstract

To understand how and why disability-free life expectancy (DFLE) trends differ by socioeconomic position (SEP) we use longitudinal data from the Cognitive Function and Ageing Studies (CFAS I: 1991; CFAS II: 2011), with two year follow up. Disability was defined as difficulty in activities of daily living, and SEP as area-level deprivation. Between 1991 and 2011, men aged 65 gained more in life expectancy (LE) than DFLE, with the greatest gain in DFLE for the most advantaged and in disability years for the most disadvantaged. The most advantaged men experienced a 60% reduction in the risk of death when disability-free, 30% reduction in incident disability, and 80% increase in recovery. The most disadvantaged experienced a 30% reduction of death but from disability. Women overall, and in the most advantaged groups, gained similar years of LE and DFLE to men but due to a 30% reduction in incident disability only.

